# Cost-effectiveness of abbreviated-protocol MRI screening for women with mammographically dense breasts in a national breast cancer screening program

**DOI:** 10.1016/j.breast.2021.12.004

**Published:** 2021-12-10

**Authors:** Jing Wang, Marcel J.W. Greuter, Karin M. Vermeulen, Frank B. Brokken, Monique D. Dorrius, Wenli Lu, Geertruida H. de Bock

**Affiliations:** a University of Groningen, University Medical Center Groningen, Department of Epidemiology, Groningen, the Netherlands; b University of Groningen, University Medical Center Groningen, Department of Radiology, Groningen, the Netherlands; c University of Groningen, Department of Computing Science, Groningen, the Netherlands; d Department of Epidemiology and Health Statistics, Tianjin Medical University, Tianjin, China

**Keywords:** Breast neoplasms, Mass screening, Breast density, Magnetic resonance imaging, Cost-benefit analysis, ACER, Average cost effectiveness ratio, AP, Abbreviated protocol, BC, Breast cancer, BI-RADS, Breast Imaging Reporting and Data System, CI, Confidence interval, DCIS, Ductal carcinoma in situ, DBT, Digital breast tomosynthesis, ICER, Incremental cost-effectiveness ratio, LYG, Life years gained, MRI, Magnetic resonance imaging, QALY, Quality-adjusted life-year

## Abstract

**Introduction:**

Magnetic resonance imaging (MRI) has shown the potential to improve the screening effectiveness among women with dense breasts. The introduction of fast abbreviated protocols (AP) makes MRI more feasible to be used in a general population. We aimed to investigate the cost-effectiveness of AP-MRI in women with dense breasts (heterogeneously/extremely dense) in a population-based screening program.

**Methods:**

A previously validated model (SiMRiSc) was applied, with parameters updated for women with dense breasts. Breast density was assumed to decrease with increased age. The base scenarios included six biennial AP-MRI strategies, with biennial mammography from age 50–74 as reference. Fourteen alternative scenarios were performed by varying screening interval (triennial and quadrennial) and by applying a combined strategy of mammography and AP-MRI. A 3% discount rate for both costs and life years gained (LYG) was applied. Model robustness was evaluated using univariate and probabilistic sensitivity analyses.

**Results:**

The six biennial AP-MRI strategies ranged from 132 to 562 LYG per 10,000 women, where more frequent application of AP-MRI was related to higher LYG. The optimal strategy was biennial AP-MRI screening from age 50–65 for only women with extremely dense breasts, producing an incremental cost-effectiveness ratio of € 18,201/LYG. At a threshold of € 20,000/LYG, the probability that the optimal strategy was cost-effective was 79%.

**Conclusion:**

Population-based biennial breast cancer screening with AP-MRI from age 50–65 for women with extremely dense breasts might be a cost-effective alternative to mammography, but is not an option for women with heterogeneously dense breasts.

## Highlights

• AP-MRI can be cost-effective for screening women with extremely dense breast.
• The more frequent the use of AP-MRI, the more life years will be gained.
• Biennial AP-MRI for women with extremely dense breast up to age 65 is optimal.

## 1 Introduction

Breast cancer is the most common cancer among women in Europe where around one in seven women will develop breast cancer during their lifetime [1]. Previous evidence has shown that regular mammography screening can reduce breast cancer mortality by approximately 23% amongst women who are invited to attend screening [2]. However, the limited sensitivity of mammography especially in women with heterogeneously or extremely dense is also well-documented [3,4]. High breast density is not only related to a limited mammographic sensitivity and a high interval-cancer rate [4,5], but also to an elevated risk of breast cancer [6,7]. Therefore, to improve the effectiveness of screening among women with dense breasts, it is important to identify possible alternatives to mammography.

Magnetic resonance imaging (MRI), as one of the screening modalities that might provide more benefits for women with dense breasts than mammography, is considered as the most sensitive technique which is not influenced by breast density [[8], [9], [10]]. Thus far, due to relatively low accessibility and high cost, screening MRI has been used only for women at high risk (e.g., carriers of gene mutations, estimated lifetime risks ≥20%) as a supplemental tool to breast screening with mammography [11,12]. However, in 2014, Kuhl et al. proposed a fast abbreviated protocol (AP) for MRI, making it possible for MRI to be used as a screening modality in a more general population [13]. Compared with a full protocol MRI, AP-MRI can remarkably reduce the associated acquisition time from 20 to 60 min to only 3–15 min, which in turn reduces the related costs while maintaining diagnostic accuracy and cancer detection [13,14].

Recent studies have shown that AP-MRI can improve the early diagnosis of breast cancer in women with dense breasts, who are at a relatively higher risk of breast cancer [15]. Although the utilization of AP-MRI showed promising results, whether it could be implemented as a cost-effective screening modality remains unknown [[16], [17], [18]]. Therefore, we aimed to investigate whether AP-MRI could be used as a cost-effective alternative to mammography in women with dense (heterogeneously or extremely) breasts in a breast cancer screening program using a microsimulation model. In addition, in this analysis, instead of assuming breast density remained constant, we modelled breast density dynamically to reflect the fact that breast density will reduce with increased age.

## 2 Methods

This study was reported according to the Consolidated Health Economic Evaluation Reporting Standards (CHEERS) statement [19]. The previously validated micro-simulation model SiMRiSc was used in this analysis [[20], [21], [22]], the full SiMRiSc code can be accessed on https://fbb-git.gitlab.io/simrisc/, or https://packages.debian.org/sid/simrisc. Based on the purpose of this study, we updated the related input parameters of the model by searching published data for women with dense breasts.

### 2.1 Model summary

In brief, women's lifetimes were simulated by considering their life expectancy, the chance of developing cancer, tumour growth, tumour self-detection probability and survival probability. Only invasive cancers were considered. If a woman did not develop a tumour and death was due to causes other than breast cancer, the chance of survival was calculated based on age-specific mortality in the general population. If a tumour developed, whether it will be screen- or self-detected depended on the sensitivity of the screening modality or probability of self-detection of the tumour. After diagnosing breast cancer, either by screening or self-detection, the breast cancer age-specific death of a woman was calculated based on life expectancy which depended on tumour size. Also, false positives were included and if ionizing radiation was applied, the probability of tumour induction was also estimated. A detailed description can be found in previously published studies [[20], [21], [22], [23]].

### 2.2 Input parameters

The estimates for the model input parameters were based on population statistics and the results of systematic searches [7,20,[24], [25], [26], [27], [28], [29], [30], [31], [32]], which are shown in Table 1. To make the SiMRiSc model applicable to MRI screening in women with dense breasts, we updated the related parameters (illustrated below) from Koleva-Kolarova et al. by searching published data for women with mammographic dense breasts [23].

**Table 1:** Input variables and their estimates for the SiMRiSc model.

	Variables		Estimates (95% CI)				Distribution	Reference
Tumour incidence model	Lifetime risk for heterogeneously dense breasts until age 75		15.5% (14.5–16.6)				Normal	7, 24-25
	Lifetime risk for extremely dense breasts until age 75		20.2% (18.8–21.6)					
	Mean onset age		67.9 (65.7–70.1)					
	Spread		21.1 (19.2–22.9)					
Tumour growth model	Tumour volume doubling time (TVDT) per age group	<50	80 (28) days				Log normal	26
		50–70	157 (25) days					
		>70	188 (52) days					
Self-detection diameter (mm)	Log-transformed mean of self-detection size		2.9 (2.8–2.9)				Log normal	27
	Spread of self-detection size		0.6 (0.4–0.7)					
Mammographic breast density	BI-RADS density distribution		a	b	c	d		
	50–60 years		0	0	0.67	0.33	–	28
	60–70 years		0	0.43	0.43	0.14		
	>70 years		0.29	0.35	0.31	0.05		
	Mean area percent density (m)		0.06	0.16	0.40	0.83	Normal	29
Tumour induction	Probability of tumour induction due to radiation per Gy		0.51 (0.28–0.83)				Normal	20
Mammography	Radiation dose (per screen) in mGy		3 (1–5)				Normal	20
	Specificity		0.89 (0.88–0.89)				Normal	30
	Sensitivity (as a function of tumour size and breast density)		Please see Supplementary data 1				–	31
AP-MRI	Sensitivity		0.95 (0.83–0.99)				Normal	15,32
	Specificity		0.87 (0.86–0.89)				Normal	

Abbreviations: CI = Confidence interval; TVDT = Tumour volume doubling time; BI-RADS= Breast Imaging Reporting and Data System; AP-MRI = Abbreviated protocol magnetic resonance imaging.

#### 2.2.1 Tumour incidence model

For the tumour incidence model, the assumption that breast cancer incidence is normally distributed as a function of age still holds for women with mammographically dense breasts, but we updated the values of lifetime risk and mean onset age because of the fundamental differences between dense breasts and non-dense breasts. The lifetime risk for breast cancer at the age of 75 for women with heterogeneously and extremely dense breasts was estimated at 15.5% and 20.2%, respectively, which was approximately 1.4 and 1.8 times larger than in the general population [24]. This updated value was estimated by considering the breast density distribution in the general population and the incidence relative risk [7,25]. As breast cancer patients with dense breasts were more likely to be diagnosed at younger ages than patients with fatty breasts [33], we estimated for the mean tumour onset age 67.9 years, which was 5 years earlier than in the general population (72.9).

#### 2.2.2 Mammographic breast density

As only women with dense breasts at age 50 were considered in this analysis, the breast density distributions were also updated. At age 50, the proportion of women with heterogeneously and extremely dense breasts were set at 67% and 33%, respectively [28]. We assumed that breast density decreased over time, so that at the age of 75 only 31% and 5% of the women had heterogeneously and extremely dense breasts, respectively [28]. The breast density distributions for different age groups are listed in Table 1.

#### 2.2.3 Test performance of mammography and AP-MRI

The test performance of mammography and AP-MRI was also searched for women with dense breasts. Unlike the previous model where mammography sensitivity depended on breast density only [23], in this analysis, the mammography sensitivity was updated by using a sensitivity function as a function of breast density as well as tumour size [31]. The sensitivity was assumed to be a logistic function of the tumour diameter (d), the mean area percentage breast density (m, scaled to [0,1]) and an interaction term m/d^2^. The area percent density values were estimated by converting the volumetric percent density for different BI-RADS groups to an area percent density [29, Table 1]. In addition, we applied a systematic error of 10% reflecting the fact that this proportion of breast tumours could not be detected by mammography due to their characteristics such as lobular carcinomas, dense breast tissue and tumours located close to the thorax wall [23]. The specificity of mammography for women with dense breasts was obtained from a meta-analysis and was estimated at 0.89 (95% confidence interval [CI]: 0.88–0.89) [30].

For AP-MRI, a literature search was performed to find appropriate estimations for sensitivity and specificity, and two studies were found that presented related data of using AP-MRI as a screening modality in women with dense breasts [15,32]. The mean estimates of sensitivity and specificity were 0.95 (95%CI: 0.83–0.99) and 0.87 (0.86–0.89), respectively.

### 2.3 Costs

The costs considered in this analysis included the costs of screening exams, biopsies and the estimated costs of breast cancer treatment based on tumour size at diagnosis (Table 2 [21,33,34]). The cost of mammography per screen was € 68, which was extracted from a national report of the Netherlands [33]. The cost of AP-MRI per screen was set as € 272, which equals to that of a full protocol one as we did not find an appropriate estimate for AP-MRI [33]. In addition, to account for additional implementation costs of a new screening modality, we applied an additional cost of € 55 per AP-MRI screening. This estimation was based on a screening participation rate of 80%, and the implementation costs for the screening organization, management costs and set-up costs for quality assurance and training [27,33]. All costs were updated to 2019 euros using the Dutch consumer price index [35].

**Table 2:** Costs of breast cancer screening, biopsy and treatment, indexed to 2019 values.

Item		Value	Reference
Mammography (per screen)		€ 68	33
AP-MRI (per screen)		€ 272	
Additional costs (per AP-MRI screen)		€ 55	
Biopsy		€ 191	34
Treatment (tumour diameter)	<20 mm	€ 6875	21
	20–50 mm	€ 7612	
	>50 mm	€ 8224	

Abbreviation: AP-MRI = Abbreviated protocol magnetic resonance imaging.

### 2.4 Base screening scenarios

**Fig. 1:**
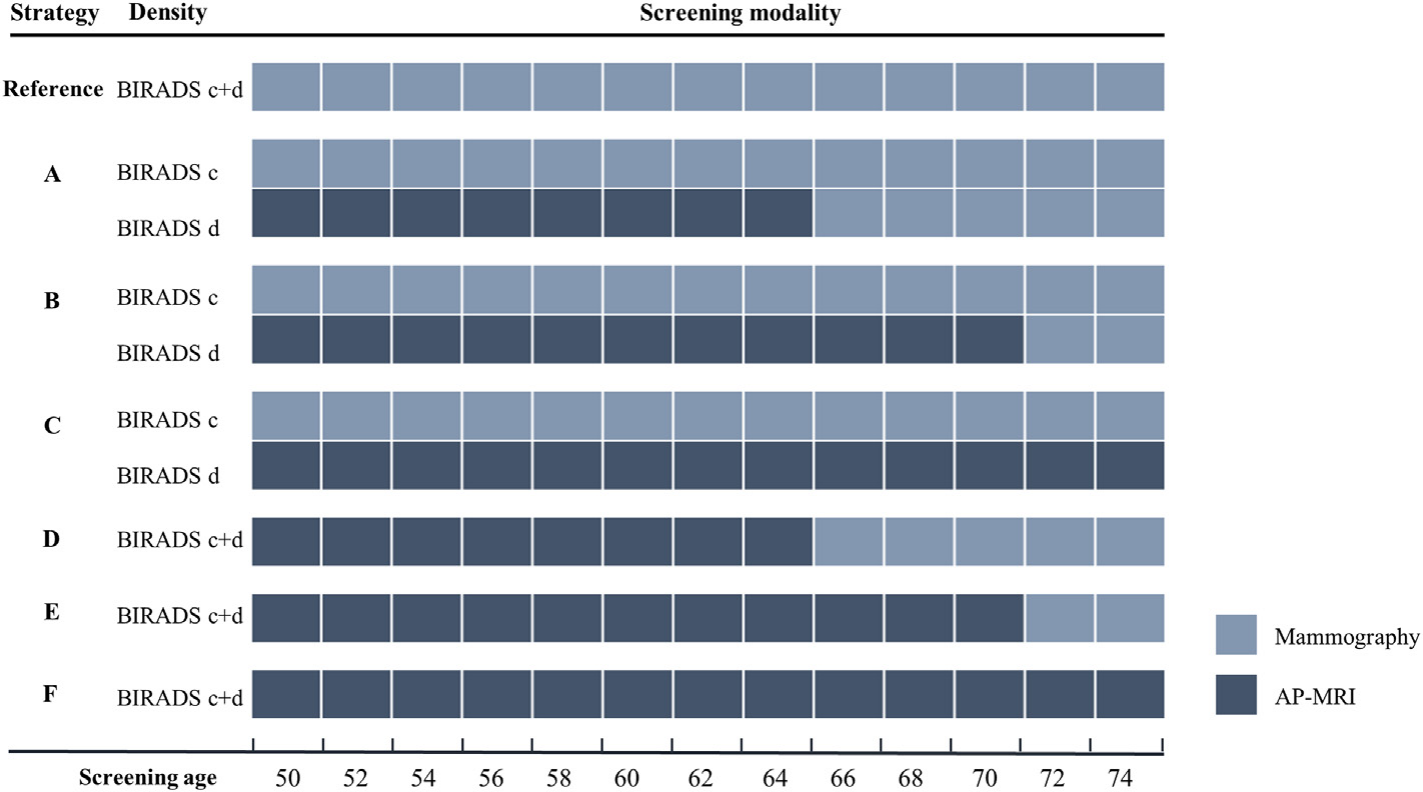
Base screening scenarios. Abbreviation: BI-RADS = Breast Imaging Reporting and Data System; BI-RADS c = heterogeneously dense, BI-RADS d = extremely dense; AP-MRI = Abbreviated protocol magnetic resonance imaging.

For base scenarios, six screening strategies (Box 1, Fig. 1) were simulated, and the current Dutch strategy for population breast cancer screening (biennial mammography from 50 to 74) was the reference [27]. A participation rate of 80% was applied in this study [27].

Box 1 **Base screening scenarios**

**Table:** 

a. Women with heterogeneously dense breasts receive biennial mammography from 50-74 years old, and women with extremely dense breasts receive biennial AP-MRI from 50-65 plus biennial mammography from 66-74 years old;
b. Women with heterogeneously dense breasts receive biennial mammography from 50-74, and women with extremely dense breasts receive biennial AP-MRI from 50-69 plus biennial mammography from 70-74 years old;
c. Women with heterogeneously dense breasts receive biennial mammography from 50-74, and women with extremely dense breasts receive biennial AP-MRI from 50-74 years old;
d. All women with dense breasts (heterogeneously and extremely dense breasts) receive biennial AP-MRI from 50-65 plus biennial mammography from 66-74 years old;
e. All women with dense breasts receive biennial AP-MRI from 50-69 plus biennial mammography from 70-74 years old;
f. All women with dense breasts receive biennial AP-MRI from 50-74 years old.

Alt-text: Box 1

### 2.5 Outcomes

We simulated 100,000 women to minimise the risk of statistical errors and to limit the computation time. Each simulation was repeated ten times to calculate the error of the point estimates, and the results were reported in terms of tumour deaths, radiation-induced tumours, screen-detected tumours, interval cancers and life years gained (LYG) per 10,000 women over their lifetimes. Average cost-effectiveness ratios (ACERs) compared to the current Dutch strategy were estimated as the ratios of the additional costs of the screening scenario to the LYG compared to the reference. In addition, incremental cost-effectiveness ratios (ICERs) were calculated based on the comparison of a lower cost scenario to the next more expensive and effective scenario after excluding dominated scenarios. A discount rate of 3% for both costs and health effects (LYG) was applied [36]. The willingness-to-pay threshold was set at € 20,000 per LYG. In addition, a discount rate of 4% for costs and 1.5% for health effects (LYG) was also applied according to the Dutch guidelines [37], and the Dutch discounted results of base scenarios are shown in Supplementary file, Table S2.

### 2.6 Alternative scenarios

In order to explore the impact of different screening intervals, twelve alternative scenarios were performed by varying the screening interval (3 or 4 years). In addition, we also applied a combined strategy of mammography and AP-MRI, in which biennial mammography from 50 to 74 plus quadrennial AP-MRI screening from 51 to 71 was applied to women with extremely dense breasts (Strategy H) or to women with heterogeneously and extremely dense breasts (Strategy I). The results of the two combined strategies are listed in Supplementary file, Table S3.

### 2.7 Sensitivity analysis

To test the robustness of our model, a univariate and probabilistic sensitivity analysis were performed. ICERs were calculated based on the comparison between the optimal strategy identified by our cost-effectiveness analysis and the reference.

For the univariate sensitivity analysis, the influence of each parameter was evaluated by its ranges specified in Table 1. In addition, the uncertainty caused by the AP-MRI cost per screening was also conducted by varying the cost by ±50%. Tornado plots were generated to visualise the impact of parameter uncertainty on the cost-effectiveness of the screening.

A probabilistic sensitivity analysis was conducted to assess the overall robustness of our model. For this, a Monte Carlo simulation with 200 iterations was performed based on the input distributions. The model overall uncertainty was estimated by a cost-effectiveness acceptability curve.

## 3 Results

### 3.1 Estimated effectiveness of AP-MRI strategies using the simulation model

Table 3 shows the estimated effectiveness of all AP-MRI strategies. For base scenarios where a two-year screening interval was applied, the use of AP-MRI screening reduced the numbers of breast cancer deaths by 2%–12% compared with the reference. Meanwhile, more screen-detected cancers (11%–66%) and less interval cancers (9%–48%) were found by AP-MRI strategies. The AP-MRI strategies produced more life years, with discounted LYG ranged from 132 to 562 per 10,000 women compared to mammography screening. The more frequent the use of AP-MRI, the more life years were gained.

**Table 3:** Effectiveness and cost-effectiveness of AP-MRI screening.

Screening strategies	BC deaths	Screen-detected cancers	Radiation-induced tumour	Interval cancers	Discounted LYG^a^	Discounted ACER^a^ (k€/LYG)	ICER (k€/LYG)
MAM 50-74	736 (3)	573 (2)	18 (0.4)	671 (2)	–	–	–
**Base scenarios**							
*Biennial screening*							
Strategy A	719 (3)	635 (2)	15 (0.4)	609 (3)	132 (2)	16.0 (0.2)	18.2
Strategy B	715 (3)	657 (2)	14 (0.4)	589 (2)	145 (1)	17.8 (0.2)	ED
Strategy C	713 (3)	674 (2)	13 (0.4)	585 (3)	149 (1)	18.8 (0.1)	ED
Strategy D	671 (2)	805 (2)	4 (0.2)	441 (2)	501 (5)	21.7 (0.2)	24.7
Strategy E	655 (2)	891 (2)	2 (0.1)	362 (1)	554 (4)	23.9 (0.2)	ED
Strategy F	649 (2)	952 (2)	NA	347 (2)	562 (5)	25.7 (0.2)	58.7
**Alternative scenarios**							
*Triennial screening*							
Strategy A-3	724 (3)	614 (2)	15 (0.4)	630 (2)	91 (1)	15.1 (0.2)	15.5
Strategy B-3	723 (3)	620 (2)	14 (0.4)	625 (2)	95 (1)	15.5 (0.2)	ED
Strategy C-3	721 (3)	634 (1)	14 (0.4)	622 (2)	98 (1)	17.0 (0.3)	ED
Strategy D-3	694 (2)	697 (1)	3 (0.2)	507 (2)	334 (7)	20.3 (0.4)	23.0
Strategy E-3	690 (2)	740 (2)	2 (0.1)	506 (2)	353 (4)	21.4 (0.3)	ED
Strategy F-3	684 (2)	790 (2)	NA	500 (2)	362 (5)	23.6 (0.3)	ED
*Quadrennial screening*							
Strategy A-4	731 (3)	586 (2)	15 (0.4)	657 (3)	52 (1)	14.7 (0.2)	14.7
Strategy B-4	729 (3)	596 (2)	14 (0.4)	649 (2)	59 (1)	17.3 (0.3)	ED
Strategy C-4	728 (3)	602 (2)	14 (0.4)	652 (3)	60 (1)	18.2 (0.3)	ED
Strategy D-4	727 (2)	567 (2)	3 (0.2)	672 (2)	158 (5)	23.4 (0.7)	ED
Strategy E-4	713 (2)	640 (2)	2 (0.1)	605 (2)	204 (5)	25.9 (0.6)	ED
Strategy F-4	711 (2)	660 (2)	NA	622 (2)	205 (5)	27.5 (0.7)	ED

Abbreviations: BC = Breast cancer; LYG = Life year gained; ACER = Average cost-effectiveness ratio; ICER = Incremental cost-effectiveness ratio; ED = Extended dominance.

a A discounting rate of 3% was applied for both costs and LYG. All data expressed as mean (SEs) per 10,000 women screened.

The results of the alternative scenarios are also shown in Table 3. When a different screening interval was applied, we found that the less frequent the screening, the less LYG. Specifically, scenarios that applied a four-year interval yielded only 52–205 LYG, which was significantly lower than for biennial scenarios. In addition, compared with the reference, the number of interval cancers for triennial and quadrennial screening scenarios reduced only by 6%–25% and 0–10%, respectively.

### 3.2 Cost-effectiveness of AP-MRI strategies

When a 3% discount rate was applied to both LYG and costs, we found that only strategies that applied AP-MRI exclusively to women with extremely dense breasts remained cost-effective, with ACERs ranging from € 14,738 to € 18,766/LYG. Given a threshold of € 20,000/LYG, strategy A, which included biennial AP-MRI from 50 to 65 plus mammography from 66 to 74, was considered optimal with an ICER of € 18,201/LYG.

### 3.3 Sensitivity analysis

**Fig. 2:**
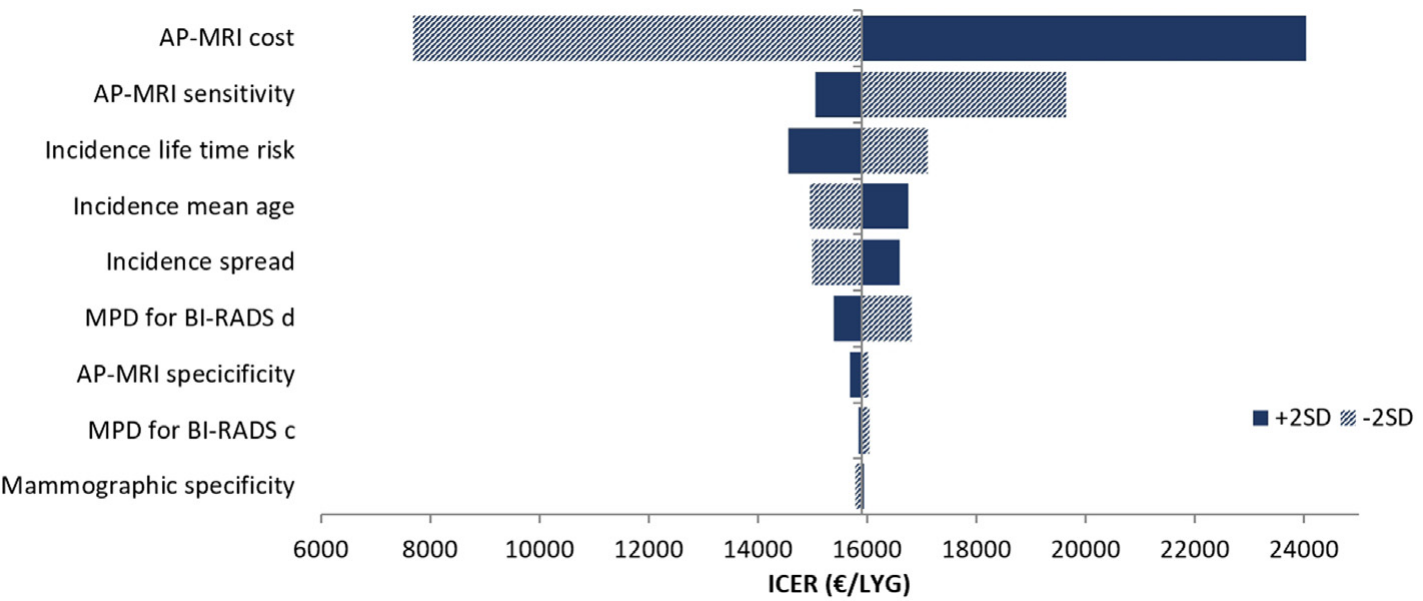
Tornado plot of the univariate sensitivity analysis. Abbreviations: AP-MRI = Abbreviated protocol magnetic resonance imaging; MPD = Mean percent density; BI-RADS= Breast Imaging Reporting and Data System; SD = Standard deviation; LYG = Life year gained; ICER = Incremental cost-effectiveness ratio.

Results of the univariate sensitivity analysis are summarized in Fig. 2. Our model was most sensitive to the cost of AP-MRI per screen, with ICERs varying from € 7758 to € 24,317/LYG. The sensitivity of AP-MRI was also an influential factor as shown in Fig. 2. In addition, parameters related to the tumour incidence model were considered as factors that moderately degrade the robustness of our model.

**Fig. 3:**
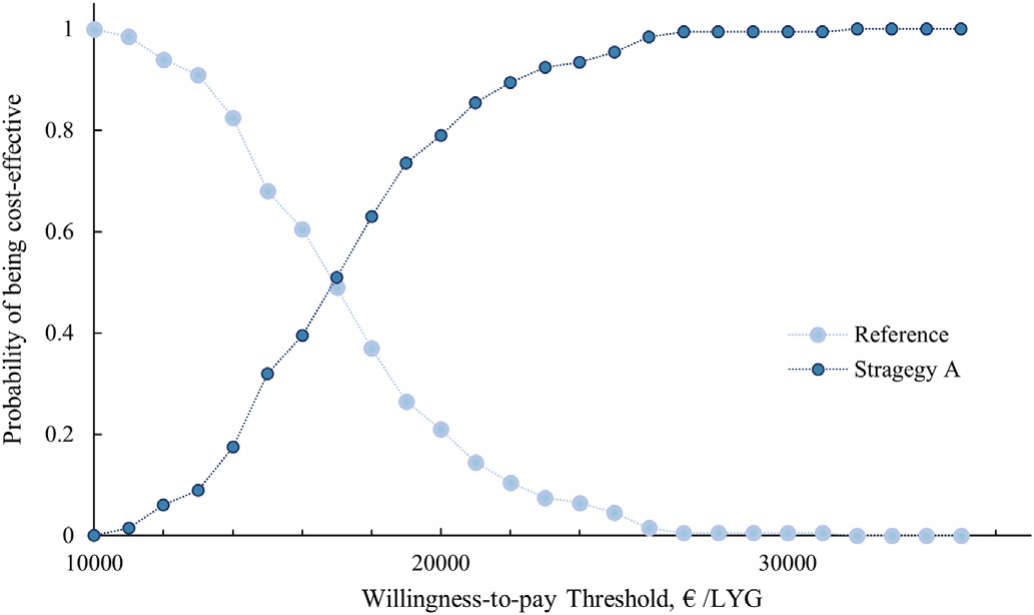
Cost effectiveness acceptability curve for the probabilistic sensitivity analysis. Abbreviation: LYG = life years gained.

**Fig. 4:**
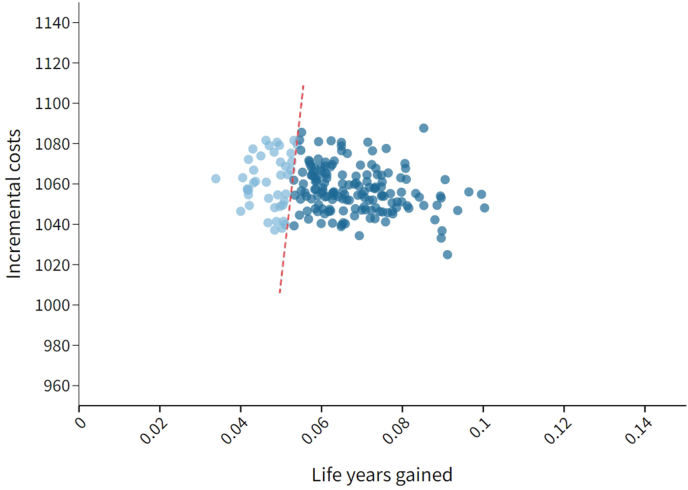
Cost effectiveness scatter plot, where light blue dots represent iterations that have an ICER larger than the threshold of € 20,000/LYG (red dashed line), and darker blue dots represent iterations that have an ICER smaller than the threshold.

Fig. 3, Fig. 4 shows the results of the probabilistic sensitivity analysis. The cost-effectiveness accessibility curve suggests that at a willing-ness to pay of € 20,000/LYG, strategy A could be a cost-effective option with a 79% probability.

## 4 Discussion

In this study we evaluated the cost-effectiveness of implementing AP-MRI in women with dense (heterogeneously or extremely dense) breasts in a population-based breast cancer screening program. We only focused on the application AP-MRI rather than a full MRI protocol as we considered that given a significantly shorter acquisition and reading time, and a comparable diagnostic accuracy [13,14], an abbreviated-protocol MRI would be more applicable in a population-based screening program. The results of base scenarios showed that compared to the reference, biennial mammography screening from age 50–74, the implementation of biennial AP-MRI in women with dense breasts improved screening effectiveness in terms of averted breast cancer deaths, screen-detected cancers and LYG. More LYG was observed in strategies with more intensive use of AP-MRI. A longer screening interval (triennial of quadrennial) yielded only limited LYG, and only a mild to moderate reduction was found in the number of interval cancers, especially for a four-year screening interval setting. In addition, our model identified that the strategy of implementing biennial AP-MRI exclusively for women with extremely dense breasts from age 50–65 was the optimal one with the highest acceptable ICER of € 18,201/LYG. The model was most sensitive to the costs of AP-MRI, followed by AP-MRI sensitivity and lifetime risk of breast cancer. The probabilistic sensitivity analysis showed that at a willing-ness to pay of € 20,000/LYG, strategy A, which used biennial AP-MRI as an alternative to mammography from age 50–65 for women with extremely dense breasts could be a cost-effective option 79% of the time.

As women with dense breasts are at an elevated breast cancer risk and are likely to be missed by mammography if a tumour is present, screening modalities other than mammography have been advocated for this group [[7], [8], [9], [10]]. In this study, we evaluated the effectiveness and cost-effectiveness of AP-MRI, one of the promising modalities, by a validated simulation model. The results elucidated that AP-MRI screening outpaces mammography screening from a long-term perspective in women with dense breasts, and screening AP-MRI could reduce breast cancer deaths by 2–12% and achieve more life-years than screening mammography, which supported the use of AP-MRI in women with dense breasts. Regarding cost-effectiveness, our results showed that AP-MRI could possibly be a cost-effective alternative to mammography in women with extremely dense breasts, with discounted ACERs ranging from € 14,738 to € 18,766/LYG. Kaiser et al. also found that MRI could be cost-effective compared to mammography, however, with a much favourable ICER of $ 8797 per quality-adjusted life-year (QALY) [38]. A probable explanation for the less favourable cost-effectiveness in our model could be that the additional costs due to implementation for screening organization, management and set-up for quality assurance and training system were included and remained constant in our model, nevertheless, on a long run, the additional costs will be less and therefore the cost-effectiveness of AP-MRI screening might be improved. In addition, Geuzinge et al. also suggested that MRI could be a cost-effective option in women with a family history of cancer [39]. However, a direct comparison could not be made as their target population of high-risk women was different from our general population.

Apart from MRI, other modalities such as ultrasound and digital breast tomosynthesis (DBT) have also been proposed to improve the screening effectiveness for women with dense breasts [[40], [41], [42]]. Ultrasound has been widely used as a supplementary method for women with dense breasts due to its easy accessibility and low costs. However, the effectiveness and cost-effectiveness of supplementary ultrasound have been questioned as several studies showed that supplementary ultrasound could only result in limited health gains at substantially increased expenses [40]. Regarding DBT, a favourable cost-effectiveness in women with dense breast was reported [22,41]. Recently, a study also compared the cost-effectiveness of DBT and AP-MRI in women with dense breasts, and demonstrated that AP-MRI could be a cost-effective alternative to DBT, at an ICER of $20,807 per QALY (at a price of $314 and $214 per screen for AP-MRI and DBT, respectively) [42]. These findings implied that if a tailored strategy for women with dense breasts is applied, AP-MRI might be the most promising alternative to mammography considering its favourable cost-effectiveness and its radiation-free feature. On the other hand, the wider use of AP-MRI might raise several practical issues related to its low accessibility and the substantial investments to initiate an AP-MRI screening program [33].

Our model was found to be most sensitive to the costs of AP-MRI per screen. This is consistent with Kaiser et al. and that the screening costs constitute a large proportion of the total costs in a population-based program with MRI as a relatively costly method [38]. The univariate sensitivity analysis of AP-MRI cost showed that with an increase of 50% for AP-MRI cost, the ICER increased to € 24,317/LYG, which was beyond the threshold of € 20,000/LYG. However, it is unlikely that the cost of AP-MRI would be more expensive than the price of a full-protocol MRI (€ 272), given that the associated acquisition time could be reduced significantly and more patients could be screened in an hour by applying the abbreviated protocol. The univariate sensitivity analysis also showed that parameters related to the tumour incidence model also had a modest impact on ICERs, which was also observed in our previous study [23]. Nevertheless, the uncertainties of these parameters did not influence the conclusion of our study as ICERs were all below the threshold.

Our study has several strengths. First, instead of assuming that breast density remained unchanged [38,39], we were able to model breast density dynamically by considering breast density distributions for different age groups. Studies that did not take breast density changes into account might lead to an underestimation of the effectiveness of mammography screening as the sensitivity of mammography increases with decreasing breast density overtime. Therefore, the cost-effectiveness of MRI screening might be overestimated in their studies. By using a breast density dependent sensitivity of mammography and considering the density changes, we estimated a more realistic cost-effectiveness of MRI with respect to mammography in women with dense breasts. Second, in this study, stratified screening strategies were performed based on BI-RADS density categories (c or d). Thus, our study could provide more tailored recommendations for women with heterogeneously and extremely dense breasts separately than other studies that took women with dense breasts as a whole (c and d) or that focused on women with extremely dense breasts only [38,42]. Third, the additional costs due to the implementation of a new screening modality were considered in this study. Because the initiation of an AP MRI-based program requires substantial investments in terms of implementation costs for screening organization, management costs and set-up costs for quality assurance and training system, we anticipate that by including these additional costs a more realistic estimation of cost-effectiveness is provided compared to some other studies [38,42].

Several limitations to this study need to be acknowledged. First, in this study, the cost of AP-MRI was assumed to be equal to the cost of a full protocol MRI (€ 272 per screen). However, as the abbreviated protocol could significantly reduce the associated acquisition time and reading time, it is likely that the cost of AP-MRI will be less expensive in the near future [13,14]. We expect that with a significantly lower price, a strategy with more intensive use of AP-MRI might be more favoured. Second, ductal carcinoma in situ (DCIS) was not included in this simulation. Previous studies have documented that MRI has a higher sensitivity for DCIS than mammography, particularly for high-grade DCIS [43]. We anticipate that by including DCIS, AP-MRI will not necessarily lead to a less favourable ICER as less aggressive and less expensive measures such as active monitoring for low-grade DCIS has been suggested by ongoing trials (for instance, the LORIS trial from UK and the LORD trial from the Netherlands), and as high-grade DCIS, which are more found by AP-MRI than mammography, have a lower risk of being overdiagnosed [44,45]. Therefore, we do not expect that the inclusion of DCIS would profoundly alter our major conclusion. Last but not least, although the effectiveness of biennial AP-MRI screening might not differ fundamentally between the Dutch screening program and many organized population screening programs practising biennial mammography screening, we need to emphasize that the costs of AP-MRI and mammography were limited to the Dutch screening setting. Therefore, considerable caution is required when extrapolating the conclusion to other countries.

## 5 Conclusion

With the development of the abbreviated protocol, MRI becomes feasible to be used in a more general population such as women with dense breasts. The results of our simulation model elucidate that the effectiveness of AP-MRI screening outpaces that of mammography screening on a long-term basis in women with dense breasts. The cost-effectiveness analysis showed that in a population-based biennial screening program, using AP-MRI as an alternative to mammography from age 50–65 for women with extremely dense breasts is cost-effective, although it is not an option for women with heterogeneously dense breasts.

## Funding

This research did not receive any specific grant from funding agencies in the public, commercial, or not-for-profit sectors.

## Declaration of competing interest

The authors declare no conflict of interest.
