# Supplementary material for: Cost-effectiveness of abbreviated-protocol MRI screening for women with mammographically dense breasts in a national breast cancer screening program
Source: Breast. 2021 Dec 10;61:58–65. doi: 10.1016/j.breast.2021.12.004 (PMC8683595; doi:10.1016/j.breast.2021.12.004)
Supplement: Multimedia component 1 [file mmc1.docx]

Supplementary file

Supplementary data 1

Mammography sensitivity model

The sensitivity function described by Isheden & Humphreys (2017) was applied in this study [19]. The sensitivity assumed a logistic function (Figure S1), depending on both tumour diameter and percentage density:

$Screening sensitivity (m,d) =\frac{1}{1+exp-\left( \beta_{1}+\beta_{2}d+\beta_{3}m+\beta_{4}\frac{m}{d^{2}} \right)}$ [Eq. (A.1)]

This function consists of the variables diameter (d) and percentage density (m, scaled to [0,1]) and an interaction term m/d^2^, which is included to capture an interplay between tumour size and area mammographic percent density (Isheden & Humphreys, 2017, Table S1). In addition, we also applied a systematic error of 0.1, which reflected that 10% of all cases that should be detected based on tumour volume could not be detected due to their characteristics such as lobular carcinomas, dense breast tissue and tumours located close to the thorax wall [23].

**Table S1** Parameter estimates of the mammography sensitivity model

| Parameters | Values (95%CI) |
| --- | --- |
| $\beta_{1}$ | -4.38 (-3.76, -3.98) |
| $\beta_{2}$ | 0.49 (0.40, 0.60) |
| $\beta_{3}$ | -1.34 (-3.00, -0.08) |
| $\beta_{4}$ | -7.18 (-16.11, -2.77) |

**Figure S1** The mammography sensitivity as a function of tumour size; BI-RADS= Breast Imaging Reporting and Data System

Supplementary data 2

**Table S2** Effectiveness and cost-effectiveness of biennial AP-MRI screening

| **Screening strategies** | **Discounted LYG^*^** | **Discounted ACER^*^ (k€/LYG)** | **Discounted ICER**  **(k€/LYG)** |
| --- | --- | --- | --- |
| Strategy A | 187 (3) | 10.8 (0.2) | 10.8 (0.2) |
| Strategy B | 204 (3) | 11.2 (0.2) | 20.6 (2.1) |
| Strategy C | 222 (1) | 11.6 (0.6) | 36.4 (14.8) |
| Strategy D | 700 (7) | 14.7 (0.1) | 15.9 (0.2) |
| Strategy E | 786 (6) | 15.7 (0.1) | 24.3 (1.8) |
| Strategy F | 802 (7) | 16.5 (0.2) | 44.9 (5.3) |

* A discount rate of 4% for costs and 1.5% for LYG was applied. All data expressed as mean (SEs) per 10,000 women screened. Abbreviations: BC = Breast cancer; LYG = Life year gained; ACER = Average cost-effectiveness ratio; ICER = Incremental cost-effectiveness ratio.

**Table S3** Effectiveness and cost-effectiveness of combined strategies

| Screening strategies | BC deaths | Screen-detected cancers | Interval cancers | Discounted LYG^a^ | Discounted ACER^a^ (k€/LYG) | ICER (k€/LYG) |
| --- | --- | --- | --- | --- | --- | --- |
| MAM 50-74 | 736 (3) | 573 (2) | 671 (2) | - | - | - |
| Strategy H^b^ | 721 (3) | 639 (2) | 609 (3) | 103 (1) | 17.5 (0.2) | ED |
| Strategy I^c^ | 678 (2) | 828 (2) | 434 (2) | 406 (5) | 22.8 (0.3) | ED |

All data expressed as mean (SEs) per 10,000 women screened.

a: A discounting rate of 3% was applied for both costs and LYG; b: Strategy H represents biennial mammography from 50-74 plus quadrennial MRI screening from 51-71 for women with extremely dense breasts; c: Strategy I represents biennial mammography from 50-74 plus quadrennial MRI screening from 51-71 for women with heterogeneously and extremely dense breasts.

Abbreviations: BC = Breast cancer; LYG = Life year gained; ACER = Average cost-effectiveness ratio. ICER = Incremental cost-effectiveness ratio; ED = Extended dominance.
